# A Novel Liver Fibrosis Marker FIB‐5 Index Predicted Response to Cardiac Resynchronization Therapy and Prognostic Outcomes in Patients With Heart Failure

**DOI:** 10.1111/anec.70004

**Published:** 2024-08-05

**Authors:** Tomoya Iwawaki, Yasuya Inden, Satoshi Yanagisawa, Takayuki Goto, Shun Kondo, Masaya Tachi, Kei Hiramatsu, Ryota Yamauchi, Masafumi Shimojo, Yukiomi Tsuji, Toyoaki Murohara

**Affiliations:** ^1^ Department of Cardiology Nagoya University Graduate School of Medicine Nagoya Japan; ^2^ Department of Advanced Cardiovascular Therapeutics Nagoya University Graduate School of Medicine Nagoya Japan

**Keywords:** cardiac resynchronization therapy, fibrosis‐4 index, fibrosis‐5 index, heart failure, liver fibrosis, responder

## Abstract

**Background:**

The fibrosis‐5 (FIB‐5) index is a noninvasive marker for assessing the progression of liver fibrosis and predictor in patients with heart failure (HF). This study investigated the association between the FIB‐5 index and response to cardiac resynchronization therapy (CRT) and evaluated its predictive value for prognosis.

**Methods:**

In total, 203 patients who underwent CRT/CRT‐defibrillator (CRT‐D) implantation were retrospectively included. The FIB‐5 index was calculated using blood samples obtained before and after CRT/CRT‐D. Response to CRT was defined as a relative reduction in left ventricular end‐systolic volume of ≥15% 6 months after CRT/CRT‐D. We compared the prognosis after CRT/CRT‐D between the groups according to the FIB‐5 index.

**Results:**

One hundred and twenty‐three patients (61%) responded to CRT. The responder group demonstrated a significantly higher FIB‐5 index than the nonresponder group (−2.76 ± 3.85 vs. −4.67 ± 3.29, *p* < 0.001). Receiver‐operating characteristic analysis demonstrated that the area under the curve of the FIB‐5 index was 0.660 with a cutoff value of −4.00 for responders. In multivariate analysis, FIB‐5 index ≥ −4.00 was an independent predictor for CRT response (odds ratio: 3.665, *p* = 0.003), in addition to QRS duration ≥ 150 ms and echocardiographic dysynchrony. The FIB‐5 index increased significantly after 6 months in the responder group but not in the nonresponder group. The FIB‐5 index ≥ −4.00 group showed a significantly better prognosis for cardiac death, HF hospitalization, and composite endpoint than the FIB‐5 index < −4.00 group.

**Conclusion:**

The FIB‐5 index in addition to classical predictors may be a useful marker for predicting response to CRT.

## Graphical abstract

Liver fibrosis and prognoses after CRT are strongly associated. The FIB‐5 index may be a useful marker for predicting response to CRT, with the potential benefit of risk stratification for the development of hospitalization for HF and all‐cause death.

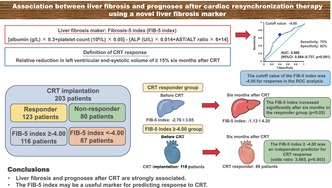

## 1 Introduction

Cardiac resynchronization therapy (CRT) is an effective treatment for patients with reduced left ventricular ejection fraction (LVEF) and prolonged QRS duration (Brignole et al. 2013). CRT improves cardiac function, alleviates heart failure (HF) symptoms, enhances exercise tolerance, and reduces mortality and HF hospitalization (Foley, Leyva, and Frenneaux 2009; Moss et al. 2009). However, these effects are predominantly observed in patients who respond to CRT therapy, and there are some, nonresponders, who do not respond to CRT, resulting in poor prognosis and increased mortality. Echocardiography is commonly used to assess the indications for CRT and evaluate the response to CRT after implantation. Liver dysfunction is an important contributor to the pathology and severity of HF, and its progression significantly impacts the outcomes of patients with coronary artery disease and HF (Itier et al. 2021; Iwawaki et al. 2023; Liu et al. 2022; Maeda et al. 2023; Nakashima et al. 2021, 2022; Shibata et al. 2021). Previous studies reported the significant value of biomarkers related to liver function (albumin and bilirubin) for predicting outcomes in patients who underwent CRT (Saito et al. 2022; Kaneshiro et al. 2021).

Liver fibrosis markers have recently been developed as a noninvasive scoring system to assess prognosis in patients with HF as well as liver disease (Cordero et al. 2023). Although the fibrosis‐4 (FIB‐4) index and nonalcoholic fatty liver disease fibrosis score (NFS) have been widely used in clinical studies so far (Iwawaki et al. 2023; Liu et al. 2022; Maeda et al. 2023; Nakashima et al. 2021, 2022; Shibata et al. 2021), an updated novel liver fibrosis marker, the fibrosis‐5 (FIB‐5) index, has been recently proposed to reflect liver fibrosis more accurately and also to predict outcomes of HF with higher sensitivity than that of the FIB‐4 index (Attallah et al. 2006; Maeda et al. 2022; Metwally et al. 2020). We hypothesized that this novel fibrosis scoring system, the FIB‐5 index, can predict response and outcomes in patients with severe HF statues who are scheduled for CRT/CRT‐defibrillator (CRT‐D) implantation. To date, few studies have evaluated the impact of liver fibrosis markers on CRT recipients (Acar et al. 2023). The aim of this study was to investigate the relationship between liver fibrosis markers and outcomes in patients who underwent CRT/CRT‐D device implantation and to evaluate the predictive value of the FIB‐5 index after CRT/CRT‐D.

## 2 Methods

### 2.1 Study Population

The study population was retrospectively recruited from a device dataset at Nagoya University Hospital, Japan. First, 241 patients who underwent CRT/CRT‐D device implantation at the Nagoya University Hospital between April 2008 and March 2020 were assessed. Twenty‐four patients with missing necessary data (mostly in echocardiographic data) were excluded from the study. Six patients with a history of chronic liver diseases, such as viral hepatitis, alcoholic hepatitis, nonalcoholic hepatitis, cirrhosis, and liver cancer, and eight patients with blood disorders were excluded from the study because these conditions could potentially affect blood test results. Moreover, we confirmed that there was no patients with active bone or tumor disease, patients <20 years of age, pregnant women, and protein‐losing diseases including nephrotic syndrome, because these pathologies may affect alkaline phosphatase (ALP) and albumin levels. In total, 203 patients were included in this study. Forty‐one patients had an upgrade to CRT from a pacemaker or an implantable cardioverter defibrillator (ICD). The indications for CRT/CRT‐D device implantation were in accordance with the guidelines (Nogami et al. 2021). The research protocol was approved by the Ethics Committee of Nagoya University Hospital. Written informed consent was obtained from all patients prior to the procedure. The study was conducted in accordance with the principles of the Declaration of Helsinki.

### 2.2 Examinations and Echocardiography

Before CRT/CRT‐D, electrocardiography, echocardiography, and blood examinations were performed in all patients. The 12‐lead electrocardiogram was performed with a paper speed of 25 mm/s and a scale of 10 mm/mV (Cardiostar FCP‐7541; Fukuda Denshi, Tokyo, Japan). The QRS duration was the maximum duration recorded for all the 12 surface leads. A complete left bundle branch block (CLBBB) was defined according to recent guidelines (Glikson et al. 2021).

For echocardiographic evaluation, left ventricular end‐diastolic volume (LVEDV) and left ventricular end‐systolic volume (LVESV) were measured using apical two‐ and four‐chamber views. LVEF was measured using Simpson's method. The left atrial diameter (LAD) was measured using the M‐mode method. The septal‐to‐posterior wall motion delay (SPWMD), left ventricular pre‐ejection period (LVPEP), and interventricular mechanical delay (IMD) were calculated as dyssynchrony parameters. We assessed i‐Index, a combined assessment of radial left ventricular (LV) dyssynchrony and contractility, using 2D speckle‐tracking strain echocardiography, as previously reported by our institution (Inden et al. 2010). The severity of mitral regurgitation and tricuspid regurgitation (TR) was assessed semiquantitatively from color Doppler images using apical two‐ and four‐chamber images (Ohte et al. 2022). Right ventricular pressure (RVP) was estimated by calculating the pressure gradient between the right atrium (RA) and right ventricular (RV) [(maximal velocity of tricuspid regurgitation)^2^ × 4] plus RA pressure.

### 2.3 CRT Implantation Procedure

CRT/CRT‐D device implantation is typically performed using the subclavian vein approach with a standard extrathoracic puncture technique. Left‐sided venous access was predominantly used as the primary approach, although in certain cases, right‐sided venous access was also selected if applicable. The RV lead was positioned within the RV septum, whereas the RA lead was placed in the RA appendage. In LV lead placement, coronary sinus angiography was performed to select an appropriate pacing site for the LV (typically in the midportion of the lateral or posterior–lateral vein), where standard pacing parameters such as threshold, sensing, and stability were acceptable without stimulating the phrenic nerve. We targeted the optimal position of the LV lead on the opposite side with respect to the RV lead position, with adequate electrical delay and anatomical distance. In some cases, the RV lead was additionally adjusted to the opposite side of the LV lead position following LV fixation, thereby maintaining sufficient distance. Device programming was performed at the discretion of the attending electrophysiologist and the clinical engineer.

### 2.4 Assessment of the Liver Fibrosis Scoring System

The FIB‐5 index was calculated based on measurement of blood aspartate aminotransferase (AST), alanine aminotransferase (ALT), ALP, platelet count, and albumin, obtained the day before the procedure. Blood sampling was performed following the Japanese Society of Laboratory Medicine guidelines (Guidelines of the Japanese Society of Laboratory Medicine (in Japanese) n.d.). None of the patients in this study had undergone recent albumin infusions or blood transfusions before the sampling day. The formula for the FIB‐5 index is [albumin (g/L) × 0.3 + platelet count (10^9^/L) × 0.05]–[ALP (U/L) × 0.014 + AST/ALT ratio × 6 + 14] (Attallah et al. 2006). For a comparison arm to the FIB‐5 index, classical fibrosis markers, the FIB4‐index and NFS were also included in the analysis. The FIB‐4 index and NFS were calculated from the same samples by the following formula: FIB‐4 index = [(age (years) × AST (U/L))/(platelet count (10^9^/L) × √ALT (U/L))] (Vallet‐Pichard, Mallet, and Pol 2006); and NFS = [−1.675 + 0.037 × age (years) + 0.09 × body mass index (kg/m^2^) + 1.13 × impaired fasting glucose/diabetes mellitus (Yes = 1, No = 0) + 0.99 × AST (U/L)/ALT (U/L)–0.013 × platelet count (10^9^/L)–0.66 × albumin (g/L)] (Angulo et al. 2007). A high FIB‐5 index indicates a low risk of liver fibrosis progression, while a low index indicates a high risk of liver fibrosis progression. Conversely, high FIB‐4 index and NFS indicate a high risk of progression of liver fibrosis, whereas low index and NFS indicate a low risk of liver fibrosis. In addition, these fibrosis markers were calculated from blood tests performed during the follow‐up period to assess changes in the values after CRT/CRT‐D.

### 2.5 Follow‐Up and Outcomes

After discharge, all patients were followed up in an outpatient clinic with device specialists in our hospital at 1, 3, and 6 months after the procedure, and every 6 months thereafter. At each visit, blood tests, 12‐lead electrocardiography, and transthoracic echocardiography were performed. Device interrogations were simultaneously performed to check the lead parameters, occurrence of arrhythmia, and therapeutic records during follow‐up. A remote monitoring system was introduced for almost all patients (>90%) who provided consent. When arrhythmia events, device treatments, or any abnormalities were noted in the remote monitoring system, patients were encouraged to visit the hospital, and additional examinations were performed to check their conditions.

The response to CRT (responders) was defined as patients with a relative reduction of LVESV of 15% or more on echocardiography at 6 months after implantation. Death, HF hospitalizations, and ICD treatments after CRT/CRT‐D were assessed in all patients using the hospital medical records.

### 2.6 Statistical Analysis

Continuous data are expressed as means ± SD or median (interquartile range), and categorical parameters are presented as numbers (percentages). Comparison of the parameters between the two groups was assessed using Student's *t*‐tests or the Mann–Whitney *U*‐test for continuous values and *χ* ^2^ tests or Fisher's exact test for categorical values. Repeated‐measures analysis of variance was used to evaluate significant differences in the means over time in the same subjects. Bonferroni correction was applied for multiple comparisons of the different time points. The Kolmogorov–Smirnov test was used to determine whether the sample data were normally distributed. Non‐normally distributed data measured more than twice for each group were compared using Friedman's test. Receiver‐operating characteristic (ROC) curve analysis was performed, and the corresponding area under the curve (AUC) was plotted. Youden's index was used to determine the best cutoff value based on the ROC curve (Youden 1950). Kaplan–Meier survival curves were used to visualize the survival rates, and the log‐rank test was performed to assess the statistical significance between the groups. Univariate logistic regression analysis was conducted to identify factors related to CRT response. Factors with a *p* < 0.05 in the univariate logistic regression analysis were included using a backward stepwise method in the multivariate model. A multivariable Cox regression model, including possible covariates, was conducted to examine the independent predictive value of the outcomes. Spearman's correlation coefficient analysis was used to assess the relationships between parameters. Statistical significance was set at *p* < 0.05.

## 3 Results

### 3.1 Baseline Patient Characteristics Between Responder and Nonresponder Groups

Of the 203 patients included in the study, 123 (61%) were responders at 6 months. The baseline characteristics of the responder and nonresponder groups are presented in Table 1. On echocardiography, the responder group had significantly smaller LAD (42.3 ± 7.9 mm vs. 46.9 ± 9.9 mm, *p* = 0.001), larger LV end‐diastolic diameter (65.9 ± 9.0 mm vs. 69.3 ± 11.2 mm, *p* = 0.028), and lower tricuspid regurgitation pressure gradient (TRPG) (28.4 ± 9.8 mmHg vs. 34.9 ± 14.5 mmHg, *p* = 0.019) than in the nonresponder group. Regarding dyssynchrony parameters, the responder group demonstrated more electromechanical dyssynchrony, including SPWMD, LVPEP, i‐Index, and QRS duration before CRT, and a higher prevalence of CLBBB than the nonresponder group. Additionally, the prevalence of hypertension and the use of angiotensin‐converting enzyme inhibitors (ACE‐Is) or angiotensin II receptor blockers (ARBs) were more common, whereas the use of loop diuretics was significantly lower in the responder group than in the nonresponder group.

**TABLE 1:** Baseline characteristics between the responder and nonresponder groups.

	Responder (*n* = 123)	Nonresponder (*n* = 80)	*p*
Age (years)	66.7 ± 12.3	66.7 ± 10.9	0.988
Male, *n* (%)	87 (70.7)	54 (67.5)	0.627
Body mass index (kg/m^2^)	22.7 ± 3.9	22.4 ± 4.0	0.544
CRT data			
CRT‐D, *n* (%)	106 (86.2)	69 (86.3)	0.989
CRT‐P, *n* (%)	17 (13.8)	11 (13.8)	0.989
Upgrade, *n* (%)	33 (26.8)	20 (25.0)	0.782
Primary prevention, *n* (%)	100 (81.3)	57 (71.3)	0.107
ICD activation, *n* (%)	45 (36.6)	32 (40.0)	0.626
LV lead location			
Anterolateral, *n* (%)	28 (22.8)	15 (18.8)	0.496
Lateral, *n* (%)	66 (53.7)	42 (52.4)	0.738
Posterolateral, *n* (%)	29 (23.5)	23 (28.8)	0.412
Laboratory data			
FIB‐5 index	−2.76 ± 3.85	−4.67 ± 3.29	<0.001
FIB‐4 index	1.85 (1.37–2.35)	2.20 (1.54–2.99)	0.001
NFS	−0.51 ± 1.18	0.18 ± 1.31	<0.001
BNP levels (pg/mL)	341.3 (161.5–505.6)	362.5 (215.4–632.1)	0.089
eGFR (mL/min/1.73 m^2^)	55.3 ± 22.6	51.1 ± 26.5	0.228
Echocardiographic data			
LVEF (%)	27.8 ± 7.8	28.4 ± 9.4	0.614
LAD (mm)	42.3 ± 7.9	46.9 ± 9.9	0.001
LVDD (mm)	65.9 ± 9.0	69.3 ± 11.2	0.028
LVDS (mm)	57.0 ± 9.5	60.0 ± 11.7	0.055
LVEDV (mL)	205.2 ± 75.8	218.8 ± 117.2	0.315
LVESV (mL)	148.0 ± 62.5	156.9 ± 85.6	0.424
MR (≥moderate), *n* (%)	37 (30.1)	23 (28.8)	0.694
TR (≥moderate), *n* (%)	17 (13.8)	20 (25.0)	0.055
TRPG (mmHg)	28.4 ± 9.8	34.9 ± 14.5	0.019
RVP (mmHg)	32.6 ± 9.2	36.2 ± 12.9	0.112
TAPSE (ms)	14.9 ± 4.8	12.5 ± 3.6	0.108
DCT (ms)	179.0 ± 74.9	187.5 ± 72.1	0.534
SPWMD (ms)	186.1 ± 84.7	121.6 ± 61.0	<0.001
SPWMD ≥ 130 ms, *n* (%)	87 (70.7)	26 (32.5)	<0.001
LVPEP (ms)	150.9 ± 34.0	136.9 ± 33.3	0.004
LVPEP ≥ 140 ms, *n* (%)	76 (61.8)	35 (43.8)	0.011
IMD (ms)	46.5 ± 26.0	39.6 ± 27.4	0.119
IMD ≥ 40 ms, *n* (%)	61 (49.6)	28 (35.0)	0.115
i‐Index	2159.0 (1324.3–3271.5)	1765.0 (885.1–2476.6)	0.045
i‐Index ≥ 2000, *n* (%)	68 (55.3)	29 (36.3)	0.006
Electrocardiogram data			
Heart rate (bpm)	74.4 ± 13.2	69.6 ± 16.0	0.021
PR duration (ms)	200.8 ± 71.7	202.9 ± 45.2	0.843
QRS axis	−16.1 ± 42.1	−7.5 ± 52.5	0.221
QRS duration before CRT implantation (ms)	170.1 ± 26.8	151.8 ± 27.4	<0.001
QRS duration ≥ 150 ms before CRT implantation, *n* (%)	102 (82.9)	35 (43.8)	<0.001
QRS duration after CRT implantation (ms)	142.4 ± 17.7	145.1 ± 18.1	0.307
CLBBB, *n* (%)	87 (70.7)	31 (38.8)	<0.001
CRBBB, *n* (%)	6 (4.9)	7 (8.8)	0.302
IVCD, *n* (%)	11 (8.9)	24 (30.0)	<0.001
RV pacing, *n* (%)	29 (23.6)	8 (10.0)	0.010
Comorbidity			
Hypertension, *n* (%)	28 (22.8)	9 (11.3)	0.028
Diabetes mellitus, *n* (%)	46 (37.4)	29 (36.3)	0.869
Atrial fibrillation, *n* (%)	23 (18.7)	24 (30.0)	0.073
Ischemic heart disease, *n* (%)	45 (36.6)	35 (43.8)	0.310
Atrioventricular block type III, *n* (%)	24 (19.5)	11 (13.8)	0.277
Medication			
ACE‐I/ARB, *n* (%)	99 (80.5)	49 (61.3)	0.004
β‐b, *n* (%)	97 (78.9)	70 (87.5)	0.101
Loop diuretic, *n* (%)	91 (74.0)	71 (88.8)	0.006
MRA, *n* (%)	76 (61.8)	54 (67.5)	0.410
Amiodarone, *n* (%)	30 (24.4)	30 (37.5)	0.052
Catecholamine or mechanical support prior to implantation	0 (0)	0 (0)	N/A
Etiology			
Cardiomyopathy, *n* (%)	57 (46.3)	32 (40.0)	0.376
Myocardial infarction, *n* (%)	22 (17.9)	22 (27.5)	0.117
Sarcoidosis, *n* (%)	5 (4.1)	7 (8.8)	0.185
Valvular heart disease, *n* (%)	8 (6.5)	5 (6.3)	0.201
Others, *n* (%)	25 (20.3)	13 (16.3)	0.469
CLBBB, *n* (%)	6 (4.9)	1 (1.3)	0.397
NYHA functional classification			
Class I, *n* (%)	1 (0.8)	0 (0)	0.421
Class II, *n* (%)	37 (30.1)	16 (20.0)	0.101
Class III, *n* (%)	50 (40.6)	39 (48.7)	0.258
Class IV, *n* (%)	35 (28.5)	25 (31.3)	0.672

*Note:* TAPSE was obtained in 27 and 15 patients in the responder and nonresponder groups, respectively.

Abbreviations: ACE‐I, angiotensin‐converting enzyme inhibitor; ARB, angiotensin II receptor blocker; BNP, brain natriuretic peptide; CLBBB, complete left bundle branch block; CRBBB, complete right bundle branch block; CRT, cardiac resynchronization therapy; CRT‐D, cardiac resynchronization therapy defibrillator; CRT‐P, cardiac resynchronization therapy‐pacemaker; DCT, deceleration time; eGFR, estimated glomerular filtration rate; FIB‐4, fibrosis‐4; FIB‐5, fibrosis‐5; ICD, implantable cardioverter defibrillator; IMD, interventricular mechanical delay; IVCD, intraventricular conduction disturbance; LAD, left atrial diameter; LV, left ventricular; LVDD, left ventricular end‐diastolic diameter; LVDS, left ventricular end‐systolic diameter; LVEDV, left ventricular end‐diastolic volume; LVEF, left ventricular ejection fraction; LVESV, left ventricular end‐systolic volume; LVPEP, left ventricular pre‐ejection period; MR, mitral valve regurgitation; MRA, mineralocorticoid receptor antagonist; NFS, nonalcoholic fatty liver disease fibrosis score; NYHA, New York Heart Association; RV, right ventricular; RVP; right ventricular pressure; SPWMD, septal‐to‐posterior wall motion delay; TAPSE, tricuspid annular plane systolic excursion; TR, tricuspid valve regurgitation; TRPG, transtricuspid pressure gradient; β‐b, beta blocker.

The responder group demonstrated significantly higher FIB‐5 index (−2.76 ± 3.85 vs. −4.67 ± 3.29, *p* < 0.001), lower FIB‐4 index (1.85 [1.37–2.35] vs. 2.20 [1.54–2.99], *p* = 0.001) and NFS (−0.51 ± 1.18 vs. 0.18 ± 1.31, *p* < 0.001) compared to the nonresponder group.

### 3.2 Changes in Liver Fibrosis Makers After CRT and Cutoff Values for the Response

**FIGURE 1:**
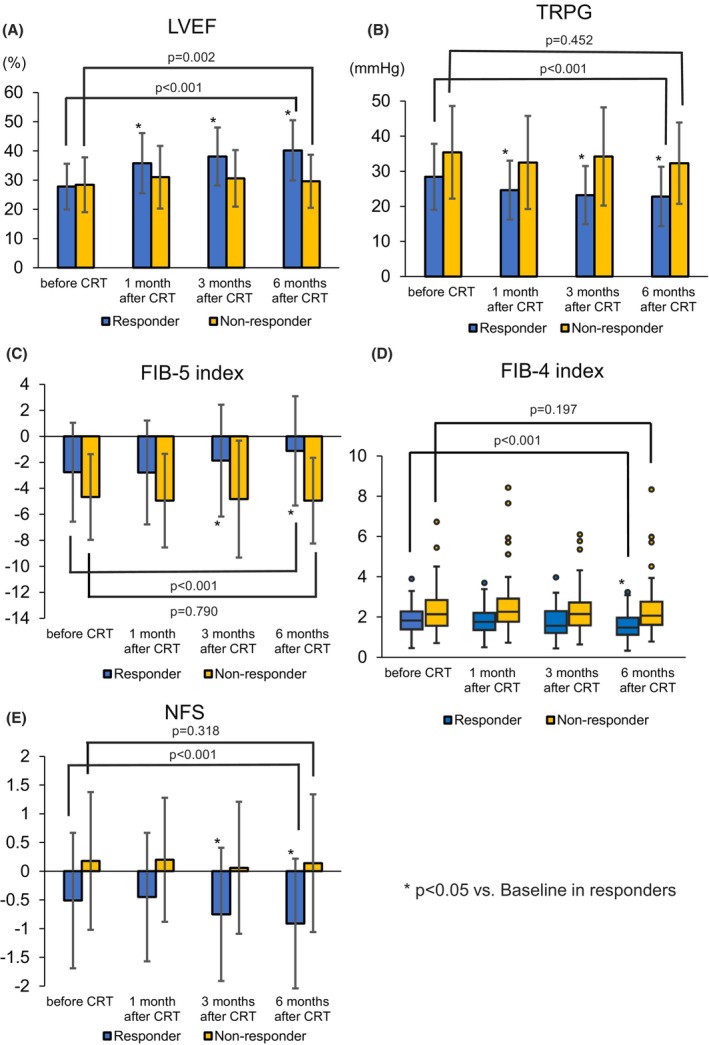
Time course of parameters including LVEF (A), TRPG (B), the FIB‐5 index (C), the FIB‐4 index (D), and NFS (E) before and after CRT/CRT‐D implantation in the CRT responder and nonresponder groups. The bar graph indicates the mean value with standard deviations, whereas the box plot indicates the median value with 1st and 3rd quantiles. Asterisks indicate multiple tests using the Bonferroni correction to compare the values before CRT with those at 1, 3, and 6 months after implantation. The *p*‐values associated with the lines indicate the overall time trend of the values from before to after 6 months using a repeated‐measures analysis of variance or Friedman's test. All values of the FIB‐5 index and NFS were normally distributed based on the Kolmogorov–Smirnov test. CRT, cardiac resynchronization therapy; CRT‐D, cardiac resynchronization therapy defibrillator; FIB‐4, fibrosis‐4; FIB‐5, fibrosis‐5; LVEF, left ventricular ejection fraction; NFS, nonalcoholic fatty liver disease fibrosis score; TRPG, tricuspid regurgitation pressure gradient.

Time‐course changes in LVEF, TRPG, the FIB‐5 index, the FIB‐4 index and NFS after CRT/CRT‐D compared between the responder and nonresponder groups are shown in Figure 1. The mean LVEF in the responder group was significantly improved from baseline to 1, 3, and 6 months after CRT/CRT‐D implantation (*p* < 0.05), while no significant difference was observed in the nonresponder group. As an overall comparison, both responder and nonresponder groups showed significant differences (responder group: *p* < 0.001, nonresponder group: *p* = 0.002) (Figure 1A). Furthermore, TRPG was significantly improved in the responder group from baseline to after 1, 3, and 6 months (*p* < 0.05), while no significant difference after CRT/CRT‐D was observed in the nonresponder group. As an overall comparison, there was a significant difference in the responder group (*p* < 0.001) (Figure 1B). In the responder group, the mean FIB‐5 index significantly increased from −2.76 ± 3.85 to −1.87 ± 4.33 and −1.12 ± 4.20 (from baseline to after three and 6 months, respectively) (*p* < 0.05). In contrast, there was no significant difference in the FIB‐5 index in the nonresponder group. As an overall comparison, there was a significant difference in the responder group (*p* < 0.001) (Figure 1C). The median FIB‐4 index significantly increased from 1.85 (1.37–2.35) to 1.47 (1.08–2.01) (from baseline to 6 months) (*p* < 0.05). In contrast, there was no significant difference in the FIB‐4 index in the nonresponder group. As an overall comparison, there was a significant difference in the responder group (*p* < 0.001) (Figure 1D). The NFS significantly decreased from −0.51 ± 1.18 to −0.75 ± 1.16 and − 0.91 ± 1.14 (from baseline to three and 6 months, respectively) in the responder group (*p* < 0.05), but did not significantly in the nonresponder group. As an overall comparison, there was a significant difference in the responder group (*p* < 0.001) (Figure 1E).

**FIGURE 2:**
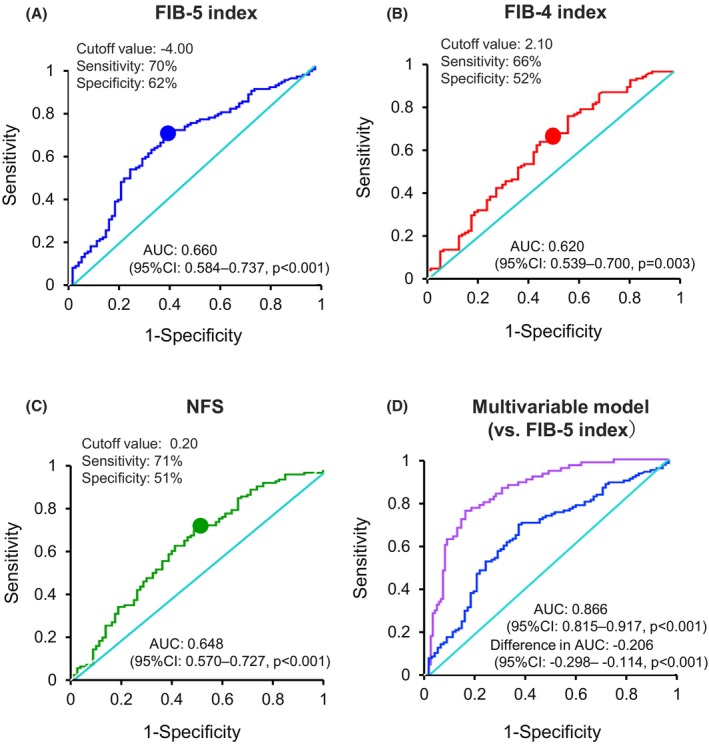
ROC curve analysis and the AUC with cutoff values for response to CRT for the FIB‐5 index (A), the FIB‐4 index (B), NFS (C), and multivariable model including all independent predictors for response to CRT (LAD, SPWMD ≥ 130 ms, LVPEP ≥ 140 ms, i‐Index ≥ 2000, QRS duration ≥ 150 ms, CLBBB, and FIB‐5 index ≥ −4.00) (D). The blue line in (D) indicates a curve of the FIB‐5 index as a reference. AUC, area under the curve; CI, confidence interval; CRT, cardiac resynchronization therapy; CLBBB, complete left bundle branch block; FIB‐5, fibrosis‐5; FIB‐4, fibrosis‐4; LAD, left atrial diameter; LVPEP, left ventricular pre‐ejection period; NFS, nonalcoholic fatty liver disease fibrosis score; ROC, receiver‐operating characteristic; SPWMD, septal‐to‐posterior wall motion delay.

The AUC of the FIB‐5 index at baseline for response to CRT was 0.660 (95% confidence interval [CI]: 0.584–0.737, *p* < 0.001) based on the ROC curve, and the cutoff value of the FIB‐5 index was determined as −4.00 with a sensitivity of 70% and a specificity of 62% (Figure 2A). In contrast, the AUC of the ROC curve for the FIB‐4 index was 0.620 (95% CI: 0.539–0.700, *p* = 0.003), and the cutoff value was 2.10 with a sensitivity of 66% and a specificity of 52% (Figure 2B). Furthermore, the AUC of the ROC curve for NFS was 0.648 (95% CI: 0.570–0.727, *p* < 0.001), and the cutoff value was 0.20 with a sensitivity of 71% and a specificity of 51% (Figure 2C).

### 3.3 Comparison of Baseline Characteristics According to the Cutoff Value of the FIB‐5 Index

The population was classified into two groups according to the cutoff value of the FIB‐5 index (FIB‐5 index ≥ −4.00 [*n* = 116], and FIB‐5 index < −4.00 [*n* = 87]). The patient characteristics of the two groups are shown in Table 2. The patients with the response to CRT were more frequent in the FIB‐5 index ≥ −4.00 group than in the FIB‐5 index < −4.00 group (86 patients [74%] vs. 37 [43%], *p* < 0.001). The FIB‐5 index ≥ −4.00 group was younger (65.1 ± 12.8 years vs. 68.9 ± 9.8 years, *p* = 0.016), and had lower brain natriuretic peptide (BNP) levels (326.7 [160.4–454.5] pg/mL vs. 428.7 [222.3–760.3] pg/mL, *p* = 0.004), TRPG (27.6 ± 10.0 mmHg vs. 34.5 ± 13.0 mmHg, *p* = 0.003), and RVP (30.9 ± 8.3 mmHg vs. 37.0 ± 11.9 mmHg, *p* = 0.004) than those in the FIB‐5 index < −4.00 group. Furthermore, the FIB‐5 index ≥ −4.00 group used more ACE‐I or ARB than the FIB‐5 index < −4.00 group (91 patients [78.4%] vs. 57 patients [65.5%], *p* = 0.045). Table S1 compares echocardiographic results 6 months after CRT/CRT‐D implantation between the FIB‐5 index ≥ −4.00 group and the FIB‐5 index < −4.00 group. The FIB‐5 index ≥ −4.00 had higher LVEF (37.5 ± 11.6% vs. 34.0 ± 10.2%, *p* = 0.018), and lower LVEDV (160.6 ± 67.6 mL vs. 191.9 ± 92.0 mL, *p* = 0.013), LVESV (106.4 ± 60.2 mL vs. 132.8 ± 80.7 mL, *p* = 0.018), and TRPG (23.0 ± 9.1 mmHg vs. 29.9 ± 11.2 mmHg, *p* = 0.001) than those in the FIB‐5 index < −4.00 group. In addition, the response rate among patients with CLBBB and a wide QRS duration > 150 ms was 85% (50 out of 59 patients) in the high FIB‐5 index group, which was higher than the 73% (22 out of 30 patients) observed in the low FIB‐5 index group (*p* = 0.234).

**TABLE 2:** Comparison of baseline characteristics between the high and low FIB‐5 index groups.

	FIB‐5 index ≥ −4.00 (*n* = 116)	FIB‐5 index < −4.00 (*n* = 87)	*p*
Age (years)	65.1 ± 12.8	68.9 ± 9.8	0.016
Male, *n* (%)	80 (69.0)	61 (70.1)	0.861
Body mass index (kg/m^2^)	22.9 ± 5.4	22.2 ± 4.2	0.288
CRT data			
CRT‐D, *n* (%)	104 (89.7)	71 (81.6)	0.113
CRT‐P, *n* (%)	12 (10.3)	16 (18.4)	0.113
Upgrade, *n* (%)	28 (24.1)	25 (28.7)	0.101
Primary prevention, *n* (%)	88 (75.9)	69 (79.3)	0.564
Responder, *n* (%)	86 (74.1)	37 (42.5)	<0.001
ICD activation, *n* (%)	41 (35.3)	36 (41.4)	0.383
LV lead location			
Anterolateral, *n* (%)	26 (22.4)	17 (19.5)	0.622
Lateral, *n* (%)	61 (52.6)	47 (54.0)	0.747
Posterolateral, *n* (%)	29 (25.0)	23 (26.4)	0.818
Laboratory data			
BNP levels (pg/mL)	326.7 (160.4–454.5)	428.7 (222.3–760.3)	0.004
eGFR (mL/min/1.73 m^2^)	52.7 ± 22.5	54.8 ± 26.4	0.543
Echocardiographic data			
LVEF (%)	27.7 ± 8.9	28.5 ± 7.8	0.492
LAD (mm)	43.2 ± 7.9	45.3 ± 10.3	0.104
LVDD (mm)	66.9 ± 9.5	67.7 ± 10.9	0.593
LVDS (mm)	57.8 ± 9.9	58.8 ± 11.2	0.494
LVEDV (mL)	204.5 ± 78.5	218.6 ± 111.9	0.292
LVESV (mL)	147.9 ± 67.4	156.4 ± 78.8	0.410
MR (≥moderate), *n* (%)	32 (27.6)	28 (32.2)	0.399
TR (≥moderate), *n* (%)	19 (16.4)	18 (20.7)	0.434
TRPG (mmHg)	27.6 ± 10.0	34.5 ± 13.0	0.003
RVP (mmHg)	30.9 ± 8.3	37.0 ± 11.9	0.004
TAPSE (ms)	14.9 ± 4.4	13.0 ± 4.5	0.163
DCT (ms)	175.5 ± 64.0	192.5 ± 83.9	0.173
SPWMD (ms)	166.1 ± 81.0	153.6 ± 84.1	0.288
SPWMD ≥ 130 ms, *n* (%)	71 (61.2)	42 (48.3)	0.068
LVPEP (ms)	146.1 ± 35.6	144.4 ± 32.8	0.730
LVPEP ≥ 140 ms, *n* (%)	65 (56.0)	46 (52.9)	0.656
IMD (ms)	42.0 ± 26.5	42.5 ± 24.9	0.883
IMD ≥ 40 ms, *n* (%)	50 (43.1)	39 (44.8)	0.662
i‐Index	2036.5 (1048.5–3190.0)	1947.0 (1219.0–3103.0)	0.439
i‐Index ≥ 2000, *n* (%)	57 (49.1)	40 (46.0)	0.348
Electrocardiogram data			
Heart rate (bpm)	72.8 ± 14.9	72.0 ± 14.1	0.682
PR duration (ms)	200.7 ± 73.0	202.8 ± 46.5	0.838
QRS axis	−13.6 ± 42.9	−11.6 ± 51.3	0.758
QRS duration before CRT implantation (ms)	165.5 ± 26.9	159.4 ± 30.1	0.133
QRS duration ≥ 150 ms before CRT implantation, *n* (%)	88 (75.9)	49 (56.3)	0.004
QRS duration after CRT implantation (ms)	143.1 ± 17.1	143.9 ± 18.9	0.773
CLBBB, *n* (%)	73 (62.9)	45 (51.7)	0.112
CRBBB, *n* (%)	7 (6.0)	6 (6.9)	0.979
IVCD, *n* (%)	18 (15.5)	17 (19.5)	0.455
RV pacing, *n* (%)	22 (19.0)	15 (17.2)	0.436
Comorbidity			
Hypertension, *n* (%)	23 (19.8)	14 (16.1)	0.497
Diabetes mellitus, *n* (%)	42 (36.2)	33 (37.9)	0.802
Atrial fibrillation, *n* (%)	24 (20.7)	23 (26.4)	0.339
Ischemic heart disease, *n* (%)	48 (41.4)	32 (36.8)	0.509
Atrioventricular block type III, *n* (%)	20 (17.2)	15 (17.2)	1.000
Medication			
ACE‐I/ARB, *n* (%)	91 (78.4)	57 (65.5)	0.045
β‐b, *n* (%)	96 (82.8)	71 (81.6)	0.833
Loop diuretic, *n* (%)	93 (80.2)	69 (79.3)	0.880
MRA, *n* (%)	68 (58.6)	62 (71.3)	0.061
Amiodarone, *n* (%)	38 (32.8)	22 (25.3)	0.245
Etiology			
Cardiomyopathy, *n* (%)	52 (44.8)	37 (42.5)	0.745
Myocardial infarction, *n* (%)	25 (21.6)	19 (21.8)	0.961
Sarcoidosis, *n* (%)	7 (6.0)	5 (5.7)	0.932
Valvular heart disease, *n* (%)	5 (4.3)	8 (9.2)	0.182
Others, *n* (%)	22 (19.0)	15 (17.2)	0.918
CLBBB, *n* (%)	5 (4.3)	3 (3.4)	0.756
NYHA functional classification			
Class I, *n* (%)	1 (0.9)	0 (0)	0.388
Class II, *n* (%)	29 (25.0)	24 (27.6)	0.680
Class III, *n* (%)	50 (43.1)	39 (44.8)	0.808
Class IV, *n* (%)	36 (31.0)	24 (27.6)	0.596

*Note:* Abbreviations are as in Table 1. TAPSE was obtained in 23 and 19 patients in the FIB‐5 index ≥ −3.95 and FIB‐5 index < −3.95 groups, respectively.

**FIGURE 3:**
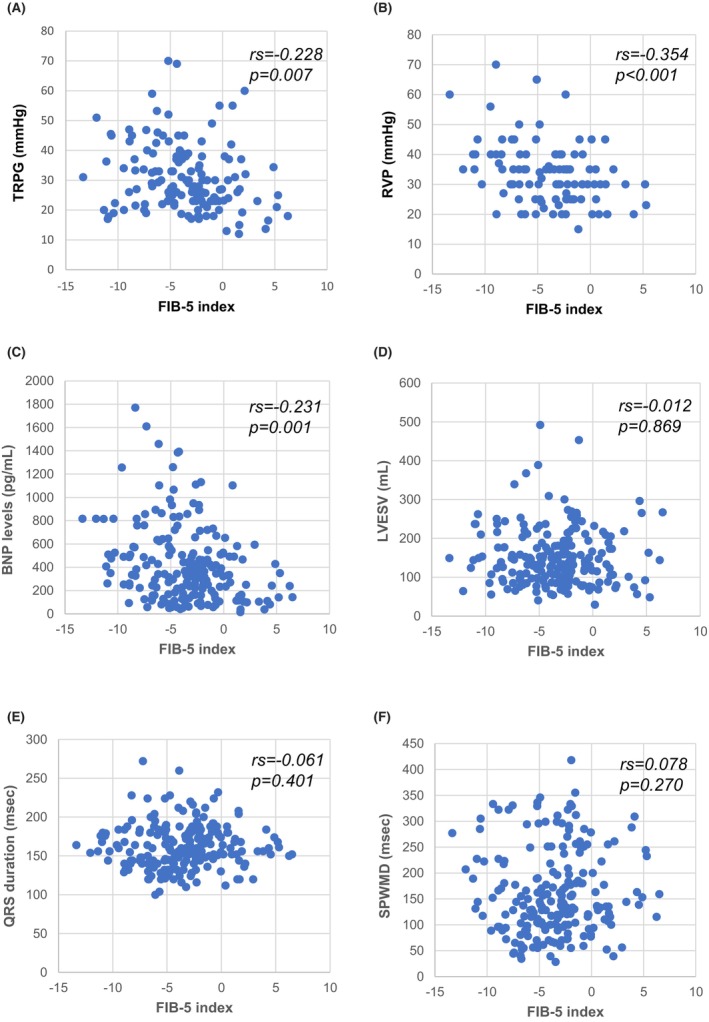
Correlations of the FIB‐5 index with TRPG (A), RVP (B), BNP levels (C), LVESV (D), QRS duration (E), and SPWMD (F). BNP, brain natriuretic peptide; FIB‐5, fibrosis‐5; LVESV, left ventricular end‐systolic volume; RVP, right ventricular pressure; SPWMD, septal‐to‐posterior wall motion delay; TRPG, tricuspid regurgitation pressure gradient.

The FIB‐5 index was significantly correlated with TRPG (*r*s = −0.228, *p* = 0.007), RVP (*r*s = −0.354, *p* < 0.001), and BNP levels (*r*s = −0.231, *p* = 0.001) at baseline (Figure 3A–C). Higher FIB‐5 index was associated with lower TRPG, RVP, and BNP levels. In contrast, there was no significant correlation between the FIB‐5 index and LVESV (*r*s = −0.012, *p* = 0.869), QRS duration (*r*s = −0.061, *p* = 0.401), or SPWMD (*r*s = 0.078, *p* = 0.270) (Figure 3D–F).

### 3.4 Predictors of CRT Response

Univariate logistic regression analysis demonstrated that LAD, SPWMD ≥ 130 ms, LVPEP ≥ 140 ms, i‐Index ≥ 2000, QRS duration ≥ 150 ms, CLBBB, ACE‐I or ARB use, amiodarone use, FIB‐4 index ≥ 2.10, NFS ≥ 0.20, and FIB‐5 index ≥ −4.00 were significantly associated with the response to CRT (Table 3). Subsequent multivariate analysis including above‐mentioned parameters showed that LAD (odds ratio [OR]: 1.048, 95% CI: 1.001–1.096, *p* = 0.046), SPWMD ≥ 130 ms (OR: 4.725, 95% CI: 2.062–10.828, *p* < 0.001), LVPEP ≥ 140 ms (OR: 2.384, 95% CI: 1.078–5.273, *p* = 0.032), i‐Index ≥ 2000 (OR: 3.599, 95% CI: 1.573–8.235, *p* = 0.002), QRS duration ≥ 150 ms (OR: 3.428, 95% CI: 1.491–7.881, *p* = 0.004), CLBBB (OR: 3.737, 95% CI: 1.660–8.417, *p* = 0.001), and FIB‐5 index ≥ −4.00 (OR: 3.665, 95% CI: 1.533–8.763, *p* = 0.003) were independent predictors of CRT response.

**TABLE 3:** Univariate and multivariate logistic regression models of CRT response.

Variables	Univariate analysis		Multivariate analysis	
	Odds ratio (95% CI)	*p*	Odds ratio (95% CI)	*p*
Age	1.000 (0.976–1.025)	0.988		
Gender (male)	1.164 (0.633–2.137)	0.625		
Body mass index	0.982 (0.925–1.042)	0.544		
BNP levels	1.001 (1.000–1.002)	0.070		
eGFR	0.993 (0.981–1.005)	0.228		
LVEF	1.009 (0.976–1.043)	0.612		
LAD	1.063 (1.027–1.101)	0.001*	1.048 (1.001–1.096)	0.046
DCT	1.001 (0.997–1.006)	0.532		
SPWMD (≥130 ms)	5.019 (2.732–9.220)	<0.001*	4.725 (2.062–10.828)	<0.001
LVPEP (≥140 ms)	2.079 (1.173–3.684)	0.012*	2.384 (1.078–5.273)	0.032
i‐Index (≥2000)	2.251 (1.250–4.053)	0.007*	3.599 (1.573–8.235)	0.002
QRS duration (≥150 ms)	6.245 (3.277–11.900)	<0.001*	3.428 (1.491–7.881)	0.004
Ischemic heart disease	0.742 (0.418–1.317)	0.308		
Cardiomyopathy	1.295 (0.732–2.520)	0.374		
CLBBB	3.820 (2.108–6.921)	<0.001*	3.737 (1.660–8.417)	0.001
Atrial Fibrillation	0.537 (0.278–1.037)	0.064		
Atrioventricular block	1.521 (0.699–3.307)	0.290		
ACE‐I/ARB	2.610 (1.385–4.917)	0.003*		
Amiodarone	0.538 (0.292–0.991)	0.047*		
FIB‐5 index (≥−4.00)	3.874 (2.138–7.020)	<0.001*	3.665 (1.533–8.763)	0.003
FIB‐4 index (≥2.10)	0.430 (0.241–0.767)	0.004*		
NFS (≥0.20)	0.360 (0.200–0.648)	0.001*		

*Note:* Other abbreviations are as in Table 1.

Abbreviation: CI, confidence interval.

* Values included in the multivariate model using a backward stepwise method.

The AUC of the ROC curve analysis for all independent variables for CRT response (LAD, SPWMD ≥ 130 ms, LVPEP ≥ 140 ms, i‐Index ≥ 2000, QRS duration ≥ 150 ms, CLBBB, and FIB‐5 index ≥ −4.00) was 0.866 (95% CI: 0.815–0.917, *p* < 0.001) (Figure 2D).

**FIGURE 4:**
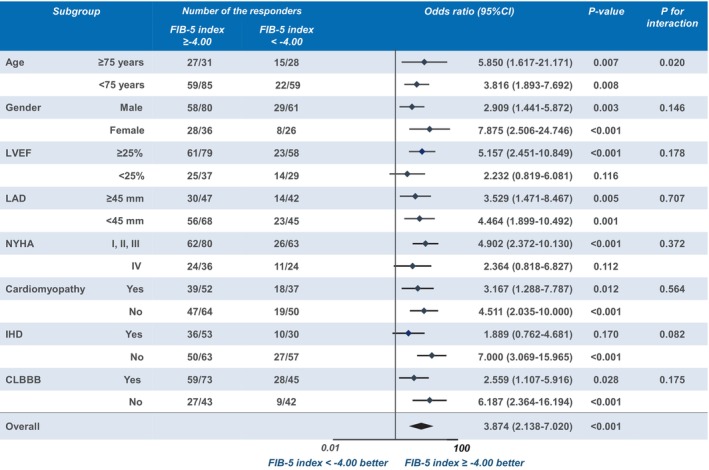
Subgroup analysis for the response to CRT between the FIB‐5 index ≥ −4.00 vs FIB‐5 index < −4.00 groups. CLBBB, complete left bundle branch block; CRT, cardiac resynchronization therapy; FIB‐5, fibrosis‐5; IHD, ischemic heart disease; LAD, left atrial diameter; LVEF, left ventricular ejection fraction; NYHA, New York Heart Association.

In the subgroup analysis for the response to CRT between the FIB‐5 index ≥ −4.00 and FIB‐5 index < −4.00 groups, the predictive ability of the FIB‐5 index was more preferable in patients with LVEF ≥ 25%, NYHA class III or less, and nonischemic heart disease compared to those without (Figure 4).

### 3.5 Clinical Outcomes After CRT by FIB‐5 Index Categories

**FIGURE 5:**
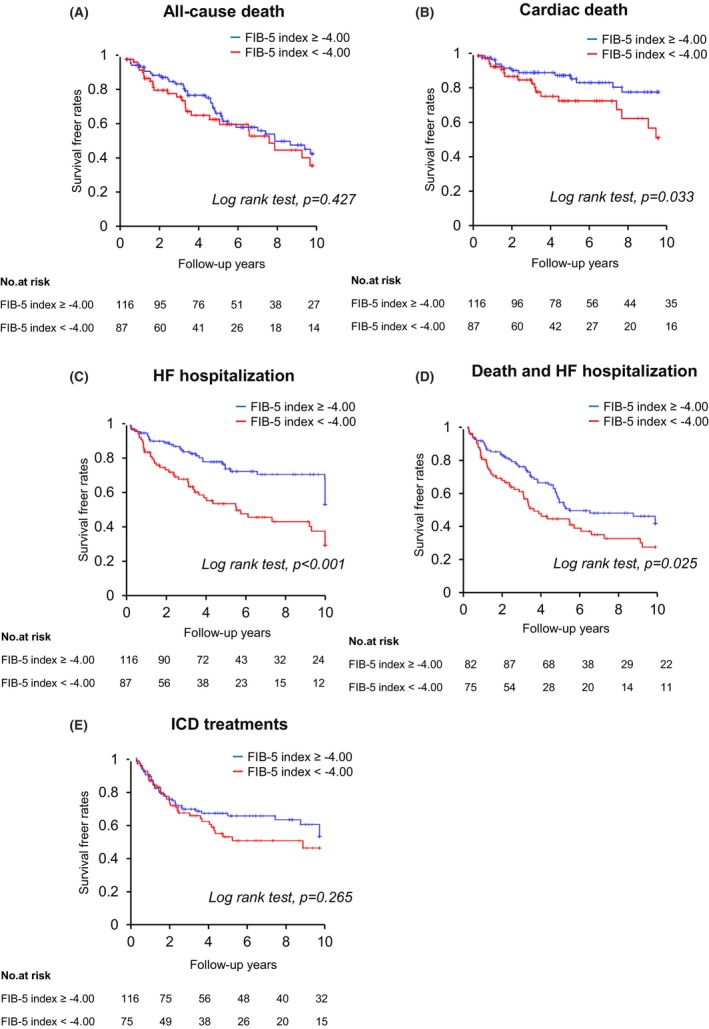
Kaplan–Meier survival curves of major adverse events after CRT/CRT‐D implantation according to the FIB‐5 index categories. (A) all‐cause death, (B) cardiac death, (C) HF hospitalization, (D) composite endpoint of all‐cause death or HF hospitalization, and (E) ICD treatment. CRT, cardiac resynchronization therapy; CRT‐D, cardiac resynchronization therapy defibrillator; FIB‐5, fibrosis‐5; HF, heart failure; ICD, implantable cardioverter defibrillator.

All‐cause death and HF hospitalization occurred in 62 and 77 patients during the mean follow‐up period of 5.3 ± 3.5 years after implantation, respectively. Thirty patients died from cardiac causes. Kaplan–Meier survival curve analysis showed no significant difference in all‐cause death (log‐rank test, *p* = 0.427) between the two FIB‐5 index groups (Figure 5A). However, as for cardiac death and HF hospitalization, the FIB‐5 index ≥ −4.00 group demonstrated significantly better prognosis than the FIB‐5 index < −4.00 group (log‐rank test, *p* = 0.033 and *p* < 0.001 in the cardiac death and HF hospitalization, respectively) (Figure 5B,C). A composite endpoint of all‐cause death and HF hospitalization occurred less frequently in the FIB‐5 index ≥ −4.00 group than in the FIB‐5 index < −4.00 group (log‐rank test, *p* = 0.025) (Figure 5D). ICD treatment (antitachycardia pacing or cardioversion/defibrillation) was not significantly different between the FIB‐5 index ≥ −4.00 and FIB‐5 index < −4.00 groups (log‐rank test, *p* = 0.265) (Figure 5E).

Multivariable Cox regression analysis, including age, BNP levels, TRPG, and ACE‐I/ARB that were shown to be different between the FIB‐5 index ≥ −4.00 and FIB‐5 index < −4.00 groups demonstrated that the FIB‐5 index < −4.00 was an independent predictor for HF hospitalization (hazard ratio 1.893, 95% CI: 1.043–3.438, *p* = 0.036) but not for cardiac death (*p* = 0.228) or composite endpoint (*p* = 0.464).

## 4 Discussion

This study investigated the prognostic value of a novel liver fibrosis marker, the FIB‐5 index, in patients with HF undergoing CRT/CRT‐D. Multivariate analysis demonstrated that the FIB‐5 index at baseline was an independent predictor of the CRT response. The FIB‐5 index improved significantly after 6 months in the responder group but not in the nonresponder group. The FIB‐5 index at baseline significantly correlated with BNP levels, TRPG, and RVP, and the FIB‐5 index changed in parallel with LVEF and TRPG after CRT/CRT‐D. The predictive ability of FIB‐5 index ≥ −4.00 for responders was preferable in the subgroups of the LVEF ≥ 25%, NYHA class III or less, and nonischemic heart disease. The FIB‐5 index ≥ −4.00 group had better prognosis for cardiac death, HF hospitalization and the composite endpoint of all‐cause death and HF hospitalization than the FIB‐5 index < −4.00 group.

HF and liver fibrosis are closely related to each other (Itier et al. 2021; Iwawaki et al. 2023; Liu et al. 2022; Maeda et al. 2023; Nakashima et al. 2021, 2022; Shibata et al. 2021). HF leads to increased pressure in the right‐sided heart system, which in turn leads to increased pressure in the systemic venous system, causing liver congestion and liver fibrosis (Samsky et al. 2013). Inversely, inflammatory cytokines, insulin resistance, and cardiotoxicity due to liver fibrosis can exacerbate HF (Itier et al. 2021). These interactions with each other may lead to a vicious cycle of HF exacerbation and liver fibrosis. Previous studies reported the association between a liver fibrosis marker, the FIB‐4 index, and prognosis in patients with acute HF and preserved LVEF: the FIB‐4 index was a reliable prognostic predictor of all‐cause death or HF hospitalization (Iwawaki et al. 2023; Maeda et al. 2023; Nakashima et al. 2021, 2022; Shibata et al. 2021). Recently, the FIB‐5 index was shown to be more specific than the FIB‐4 index in differentiating liver fibrosis (Attallah et al. 2006; Metwally et al. 2020), and the FIB‐5 index was reported to be a better predictor for outcomes in HF than the FIB‐4 index (Maeda et al. 2022). The FIB‐4 index incorporates age, AST, ALT, and platelet count for its calculation (Vallet‐Pichard, Mallet, and Pol 2006). The FIB‐5 index, on the other hand, excludes age from the calculation and includes AST, ALT, platelet count, albumin, and ALP (Attallah et al. 2006). Because albumin is produced in the liver, the albumin level is especially important to estimate the severity of chronic liver disease and liver capacity. Changes in albumin levels are not specific for liver disease, but are also affected by a variety of pathological conditions, including undernutrition and protein‐leakage disease, and albumin levels have been shown to be a significant predictor of adverse outcomes in patients with HF (Horwich et al. 2008; Prenner et al. 2020). In contrast, serum ALP reflects the condition and severity of liver disease and has also been reported to be a significant independent predictor of morbidity and mortality in patients with HF (Charach et al. 2021). Because of the involvement of the two above‐mentioned additional parameters, the FIB‐5 index may be a more powerful marker for predicting liver fibrosis as well as HF states than the FIB‐4 index. Consistently, our results demonstrated the independent predictor of the FIB‐5 index after the multivariate analysis in patients with severe HF receiving CRT/CRT‐D implantation, indicating that the updated fibrosis marker may be useful for predicting the outcomes of HF.

In addition to the classical parameters of CRT response, including pre‐QRS duration and echocardiographic dyssynchrony, the FIB‐5 index was found to be an independent predictor in the present study. Although a recent study demonstrated a significant association between CRT response and liver fibrosis using a classical fibrosis marker of NFS (Acar et al. 2023), the NFS did not remain an independent factor for the response in multivariate analysis in our study. The FIB5 index may be more powerful indices to predict response to CRT/CRT‐D in severe HF rather than the NFS. The high baseline FIB‐5 index group also had fewer composite endpoints (all‐cause death and HF hospitalization) than the low FIB‐5 index group in our study. The FIB‐5 index value may represent the HF condition from a specific perspective. Because the FIB‐5 index is calculated based on liver function, it may be plausible that the abnormality of this liver fibrosis marker indicates the presence of right‐sided HF rather than left‐sided HF. This hypothesis is consistent with our results showing that the FIB‐5 index was significantly correlated with TRPG and RVP but not with LVESV, suggesting that the FIB‐5 index is more directly linked to the pathology of right heart dysfunction. It is well known that HF complicated with both left‐ and right‐sided HF has poor prognosis and less therapeutic response against treatments compared to left‐sided HF only (Ghio et al. 2001; Voelkel et al. 2006). As for CRT/CRT‐D recipients, previous studies demonstrated that HF involving right heart dysfunction had poorer response to CRT treatment (Damy et al. 2013; Leong et al. 2013). The FIB‐5 index, as a possible marker of involvement of right‐sided HF, may be a useful predictor of CRT response compared to other markers of right heart function such as TR and RVP, and the traditional HF marker of BNP level, because the index combines several individual biomarkers in the complex calculation.

Changes in the FIB‐5 index according to the response to CRT may indicate that the fibrosis marker could be a timely, fluctuating biomarker representing the HF condition and therapeutic response. Although liver fibrosis is usually irreversible, liver congestion caused by HF collapse might improve, thereby the fibrosis index could change according to HF condition. Previous studies also demonstrated that CRT improved the right heart dysfunction as well as left heart dysfunction (Bleeker et al. 2005; Sharma et al. 2016), and changes in the FIB‐5 index may indirectly represent the improvement of right heart dysfunction after CRT/CRT‐D. The FIB‐5 index is clinically useful, with a simple calculation based on a blood test only, which is less time‐consuming than other echocardiography examinations, and better quantifies CRT response based on biomarkers. As a fibrosis marker, the FIB‐5 index may have potential utility in the follow‐up of patients after CRT/CRT‐D and in estimating the extent of CRT response, and provide a further perspective on the therapeutic approach or decision‐making based on its sensitivity to HF.

### 4.1 Study Limitations

This retrospective study was conducted at a single institution. The results of our study need to be validated on a larger scale. The institution had a relatively small number of patients receiving CRT/CRT‐D over the 12‐year study period. There may be inherent risks of selection bias and confounding factors that were not adequately controlled for in this study. More than 30 patients were excluded from the analysis, primarily because of missing echocardiographic data, which may have affected the outcomes. Furthermore, this study also excluded patients with chronic liver disease and those whose blood values could be affected directly by active blood diseases and other possible pathologies. However, we did not completely exclude the possibility of unrecognized diseases and unknown conditions that could influence liver function. The present study evaluated liver fibrosis through blood examination only and did not use imaging studies or liver biopsy. Although we included as many possible confounders for response to CRT as possible in the logistic regression models, there might be unknown parameters that could be associated with CRT response. The response to CRT may be affected by the baseline condition as well as various pathologies and follow‐up events after CRT/CRT‐D device implantation, including the occurrence of arrhythmia, medication approach, or development of anemia. The moderate but not strong predictive value of the AUC for the FIB‐5 index might also support the hypothesis that multiple pathologies and conditions may mutually contribute to the CRT response. However, it is important to estimate the response to CRT and future directions for treatments preoperatively, and the FIB‐5 index may have a potential benefit and utility in predicting the extent of response and HF at follow‐up.

## 5 Conclusion

The novel liver fibrosis marker, the FIB‐5 index, proved to be an independent predictor of CRT response in patients with HF. The FIB‐5 index changed according to the effect of CRT/CRT‐D treatment, with the potential benefit of risk stratification for the development of hospitalization for HF and all‐cause death. Combining various predictors including the FIB‐5 index would be important to predict the response after CRT/CRT‐D.

## Author Contributions

T.I. and S.Y. designed the study and wrote the manuscript. T.I collected and analyzed the data. T.I., Y.I., S.Y., T.G., S.K., M.T., K.H., R.Y., and M.S. carried out the examination. S.Y. and Y.I. helped the data interpretation and commented the manuscript. Y.T. and M. T. supervised this work. All authors reviewed and approved the manuscript.

## Ethics Statement

This study was approved by the local institutional ethics committee of Nagoya University Hospital.

## Consent

All patients provided written informed consent prior to the procedure.

## Conflicts of Interest

Dr. Yanagisawa is affiliated with a department‐sponsored by Medtronic Japan. Other authors have no conflict of interest.

## Supporting information


**Table S1.** Comparison of echocardiographic results between the high FIB‐5 index group and the low FIB‐5 index group 6 months after CRT implantation.

## Data Availability

The data that support the findings of this study are available from the corresponding author upon reasonable request.
