# Supplementary material for: A Novel Liver Fibrosis Marker FIB‐5 Index Predicted Response to Cardiac Resynchronization Therapy and Prognostic Outcomes in Patients With Heart Failure
Source: Ann Noninvasive Electrocardiol. 2024 Aug 5;29(5):e70004. doi: 10.1111/anec.70004 (PMC11299164; doi:10.1111/anec.70004)
Supplement: Supplementary file 1 — Table S1. Comparison of echocardiographic results between the high FIB‐5 index group and the low FIB‐5 index group 6 months after CRT implantation. [file ANEC-29-e70004-s001.docx]

**Supplemental Material**

Iwawaki *et al.*: A novel liver fibrosis marker FIB-5 index predicted response to cardiac resynchronization therapy and prognostic outcomes in patients with heart failure

**Table of contents**

***Page 2: Supplemental Table 1.*** Comparison of echocardiographic results between the high FIB-5 index group and the low FIB-5 index group 6 months after CRT implantation

**Supplemental Table 1.** Comparison of echocardiographic results between the high FIB-5 index group and the low FIB-5 index group 6 months after CRT implantation

|  | *FIB-5 index ≥-4.00*  *(n=116)* | *FIB-5 index <-4.00*  *(n=87)* | *P value* |
| --- | --- | --- | --- |
| LVEF (%) | 37.5±11.6 | 34.0±10.2 | 0.018 |
| LVDD (mm) | 61.1±12.3 | 63.2±12.1 | 0.284 |
| LVDS (mm) | 50.9±13.7 | 53.4±13.2 | 0.244 |
| LVEDV (mL) | 160.6±67.6 | 191.9±92.0 | 0.013 |
| LVESV (mL) | 106.4±60.2 | 132.8±80.7 | 0.018 |
| TRPG (mmHg) | 23.0±9.1 | 29.9±11.2 | 0.001 |
| SPWMD (msec) | 55.5±42.4 | 56.5±39.1 | 0.885 |
| LVPEP (msec) | 134.2±35.5 | 138.1±34.9 | 0.295 |
| IMD (msec) | 30.5±18.6 | 32.3±16.6 | 0.727 |
| i-Index | 1154.5  (474.0–2427.0) | 1151.5  (477.4–1770.0) | 0.105 |

CRT, cardiac resynchronization therapy; FIB-5, fibrosis-5; LVEF, left ventricular ejection fraction; LVDD, left ventricular end-diastolic diameter; LVDS, left ventricular end-systolic diameter; LVEDV, left ventricular end-diastolic volume; LVESV, left ventricular end-systolic volume; TRPG, transtricuspid pressure gradient; SPWMD, septal-to-posterior wall motion delay; LVPEP, left ventricular pre ejection period; IMD, interventricular mechanical delay
